# Elevated cerebral oxygen extraction in patients with post-COVID conditions

**DOI:** 10.1515/nipt-2024-0014

**Published:** 2024-11-20

**Authors:** Peiying Liu, Thomas Ernst, Huajun Liang, Dengrong Jiang, Eric Cunningham, Meghann Ryan, Hanzhang Lu, Shyamasundaran Kottilil, Linda Chang

**Affiliations:** Department of Diagnostic Radiology & Nuclear Medicine, 12264 University of Maryland School of Medicine, Baltimore, MD, USA; Department of Neurology, Johns Hopkins University, Baltimore, MD, USA; Department of Radiology, Johns Hopkins University, Baltimore, MD, USA; Department of Medicine, Institutue of Human Virology, University of Maryland School of Medicine, Baltimore, MD, USA

**Keywords:** Oxygen extraction fraction, COVID-19, MRI, brain, post-COVID conditions

## Abstract

**Objectives:**

Dysfunction of cerebral microcirculation due to SARS-CoV-2 infection has been postulated to be a plausible mechanism for the neurological symptoms of post-COVID-19 conditions (neuro-PCC), affecting oxygen homeostasis in the brain. In this study, we aimed to investigate the balance between cerebral oxygen delivery and consumption, measured by oxygen extraction fraction (OEF), in patients with neuro-PCC.

**Methods:**

25 participants with neuro-PCC (8 previously hospitalized and 17 not hospitalized) and 59 age-matched healthy controls were studied. Global OEF was quantified using TRUST MRI and compared across the three groups. Associations between OEF and neurobehavioral measures were also evaluated in participants with neuro-PCC.

**Results:**

OEF was significantly different (one-way ANCOVA-p=0.046) among the three groups, after accounting for age and sex. On post-hoc analyses, previously hospitalized neuro-PCC participants had significantly higher OEF (42.40 ± 5.40 %) than both uninfected controls (37.70 ± 5.09 %, p=0.032) and neuro-PCC participants without hospitalization (37.02 ± 5.05 %, p=0.015). Within the participants with neuro-PCC, OEF was significantly associated with locomotor function assessed with the 4-m walk gait speed score (β=−0.03, r=0.34, p=0.003).

**Conclusions:**

Participants with neuro-PCC had altered cerebral OEF, which is also associated with slower locomotion. OEF is a promising marker for studying neuro-PCC.

## Introduction

Since the emergence of the pandemic in November of 2019, more than 651 million COVID-19 cases were documented worldwide [1]. Among the COVID-19 survivors across the world, up to 40 % of the survivors experienced long-term effects after the acute infection [2], now known as post-COVID conditions (PCC) or long COVID. Neuropsychiatric symptoms, including inability to concentrate (described as “brain fog”), difficulty with memory, headaches, loss or change of smell and/or taste sensations, fatigue, anxiety, and depression [3, 4], are often reported in patients with PCC; these symptoms may last up to two years after the initial COVID-19 [3]. Neuropsychiatric symptoms of PCC (neuro-PCC) can be present even in those with mild COVID-19 symptoms [3], but the prevalence is much higher in hospitalized (50–70 %) compared to non-hospitalized patients (10–30 %) [1, 2].

Despite the high prevalence and persistent neurological sequelae of PCC, little is known regarding its etiology(ies) or neuropathophysiology. Various mechanisms have been proposed, including direct or indirect invasion of the virus into the brain with persistent low-grade infection, immune dysregulation, hormonal disturbances, or persistent neuroinflammation with elevated cytokine levels [4]. One potential etiology is the dysfunction of cerebral microcirculation due to SARS-CoV-2 infection [5], which may affect oxygen delivery and consumption in the brain. Oxygen delivery is often characterized by cerebral blood flow (CBF), and recent studies demonstrated CBF changes in patients with PCC [6, 7]. However, little is known about changes in oxygen consumption in patients with neuro-PCC.

The oxygen extraction fraction (OEF) is an important hemodynamic parameter reflecting the balance between oxygen consumption and delivery in the brain, and has been shown to be a sensitive marker of brain function and tissue viability in several neurodegenerative diseases [8, 9]. Therefore, we aimed to investigate OEF changes associated with neuro-PCC. To achieve this goal, we utilized a T2-relaxation-under-spin-tagging (TRUST) MRI technique [10] to assess OEF non-invasively. The preliminary findings of this work was presented in a conference in 2022 [11].

## Materials and methods

### Participants

Our Institutional Review Board approved this Health Insurance Portability and Accountability Act–compliant study, and all data were obtained with the participants’ written informed consent. All participants with neuro-PCC had documented prior COVID-19 and had at least one new cognitive or neuropsychiatric symptom after COVID-19 (i.e., memory complaints, headache, “brain fog”, loss of taste or smell, fatigue, depression or anxiety, sleep disturbances, pain). Control participants were included only if they were in good health and never had a history of COVID-19 or symptoms related to the illness. Participants who received the COVID vaccine were at least 7 days from their last dose to avoid any confounding post-vaccination sequelae.

All participants with neuro-PCC were evaluated with the NIH Toolbox^®^ (NIHTB) Cognitive Battery and Motor Battery [12]. Based on the previous findings that participants with neuro-PCS had poorer motor function, including poorer locomotion, endurance and dexterity, but relatively normal cognitive function, we focused on evaluating the relationship between OEF and motor function measures in this study, specifically, endurance/locomotion measured by gait speed of 4-m walk, and dexterity measured by pegboard-dominant hand.

### MRI and image processing

All participants underwent an MRI session on 3T Siemens Prisma MRI (Siemens Healthcare, Erlangen, Germany). Global venous oxygenation (Y_v_) was measured non-invasively with the TRUST MRI technique at the superior sagittal sinus to calculate the OEF [10]. The TRUST technique uses the spin-label principle on the venous side to separate pure venous blood and measure its T2, and then convert T2 to Y_v_ using a calibration plot based on the well-known relationship between T2 and Y_v_ [13]. This technique has been validated [13], and shown excellent reproducibility within subjects [14] and across sites [15] and MRI vendors [16]. Imaging parameters of the TRUST sequence were: repetition time (TR)=3,000 ms, echo time (TE)=3.61 ms, inversion time (TI)=1,022 ms, flip angle=90°, field of view (FOV)=220 × 220 × 5 mm^3^, voxel size=3.44 × 3.44 × 5 mm^3^, four effective TEs (1, 40, 80, and 160 ms) with a τCPMG of 10 ms, labeling thickness=100 mm, and scan duration=1.2 min. The imaging slice was positioned parallel to the anterior-commissure posterior-commissure line at 20 mm above the sinus confluence. The TRUST data were processed following the procedure described previously [14]. Briefly, after pairwise subtraction between control and label images, a preliminary region of interest (ROI) was manually drawn to include the superior sagittal sinus, and the 4 peak voxels in the ROI were chosen as the final mask for spatial averaging. The averaged venous blood signals were used to fit a monoexponential model to obtain T2, which was in turn converted to Y_v_ via a calibration plot [13]. Once Y_v_ value was obtained, the OEF was calculated as $OEF=\left(Y_{a}-Y_{v}\right)/Y_{a}\times 100\;\%$ , where Y_a_ is arterial oxygenation (assumed to be 98 %).

In participants with PCC, white matter hyperintensities (WMHs) were assessed using a two-dimensional (2D) fluid-attenuated inversion recovery (FLAIR) MRI scan with the following parameters: TR=11,000 ms, TI=2,800 ms, TE=100 ms, flip angle=90°, voxel size=1 × 1 × 2 mm^3^, 69 axial slices, and scan duration=3 min 18 s. A 3D T1-weighted Magnetization-Prepared Rapid Gradient-Echo (MPRAGE) image was also acquired with the following parameters: TR=6.5 ms, TI=843 ms, TE=3.11 ms, turbo factor=240, shot interval=3,000 ms, flip angle=8°, voxel size=1 × 1 × 1 mm^3^, FOV=240 × 256 × 204 mm^3^, and scan duration=5 min 59 s. WMH volume was quantified using an Bayesian-based automatic detection method described previously [17] using both the FLAIR image and MPRAGE image. The WMH volume was then log-transformed for statistical analyses to reduce the skewness of the original population variance.

### Statistical analysis

Cross-sectional analysis using linear regression model was performed within the entire group of participants to test for the group difference in OEF, where OEF was the dependent variable, the group was the categorical independent variable, and age and sex were covariates. Within neuro-PCC participants, regression analyses were also performed to study the correlation between OEF and motor function scores (i.e., gait speed of 4-m walk, and pegboard-dominant hand) after adjusting for age and sex. A p-value of less than 0.05 was considered statistically significant.

## Results

The characteristics of the 84 study participants are shown in Table 1. Participants were divided into three groups: 8 with neuro-PCC hospitalized due to SARS-CoV-2 infection during the acute infection phase, 17 with neuro-PCC not hospitalized during their SARS-CoV-2 infection, and 59 age-matched healthy controls without prior SARS-CoV-2 infection. Age and sex among the three groups were not different (p>0.12, Table 1). Days since COVID-19 diagnosis between the neuro-two neuro-PCC groups, with and without hospitalization, were also not different (p=0.63, Table 1).

**Table 1:** Demographic information of the participants.

	Healthy Controls	Participants with neuro-PCC, not hospitalized	Participants with neuro-PCC, hospitalized	p-Value
**Number of subjects**	59	17	8	–
**Age (years)**	38.9 ± 15.5 (23–77)	39.9 ± 12.2 (21–63)	45.6 ± 13.6 (28–63)	0.48^a^
**Gender (#M/#F)**	28/31	4/13	5/3	0.12^a^
**# COVID vaccinated/Unvaccinated/**	20/38/1	12/5/0	2/6/0	–
**Unknown**				
**Comorbid medical conditions**				
Hypertension	3	3	3	–
Pre-diabetic/Diabetes	0	0/1	1/0	–
COPD	0	2	2	–
Hyperlipidemia	0	2	1	–
Overweight/Obese	6/8	6/6	2/5	–
**Days since COVID-19 diagnosis**	–	212 ± 152	244 ± 166	0.63^b^
**Symptoms during acute COVID**				
# Sore throat (%)	–	11(64.7)	3 (37.5)	–
# Dry cough (%)	–	13 (76.5)	7 (87.5)	–
# Shortness of breath (%)	–	13 (76.5)	7 (87.5)	–
# Congestion (%)	–	14 (82.4)	6 (75.0)	–
# Fever (%)	–	14 (82.4)	7 (87.5)	–
# Palpitations (%)	–	12 (70.6)	5 (62.5)	–
# Chest pain (%)	–	9 (52.9)	6 (75.0)	–
# Nausea/Vomiting (%)	–	6 (35.3)	5 (62.5)	–
# Anorexia (%)	–	10 (58.8)	4 (50.0)	–
# Diarrhea (%)	–	7 (41.2)	5 (62.5)	–
# Pale colorations (%)	–	6 (35.3)	1 (12.5)	–
**Acute COVID treatment** ^g^				
# Days hospitalized	–	0	12.6 ± 7.8 (4–28)	–
Nasal Cannula O_2_/Hi-Flow/Ventilation/ECMO	–	0/0/0/0	5/2/2/1	–
Steroid^c^/Remdesivir/Monoclonal antibody^d^/	–	8/0/3/0/3	6/4/0/1/2	–
Anticoagulant^e^/Other^f^				

Data are presented as Mean ± SD, or n. ^a^ANOVA test. ^b^two-sample *t*-test. ^c^Dexamethasone (decadron), prednisone (rayoss), methylprednisolone (medrol) or hydrocortisone. ^d^Tocilizumab (actemra), bamlanivimab, or etesevimab. ^e^Eloquis. ^f^Azithromycin, Z pack, ceftriaxone. ^g^Hospitalized participants may have received multiple forms of treatment, i.e., ECMO, ventilation and NCO2.

**Figure 1:**
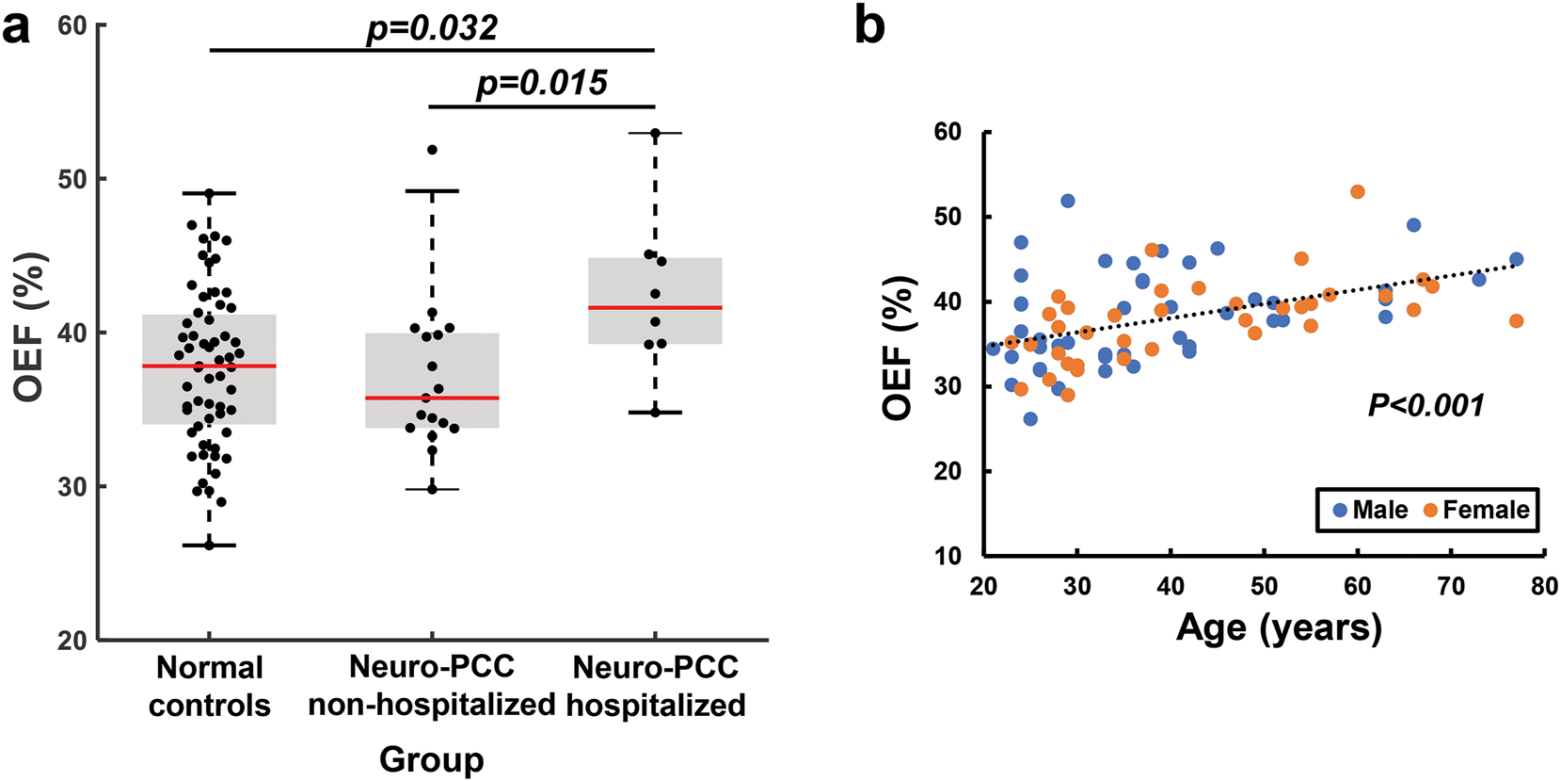
Global OEF varied across different groups and ages. (a) Comparisons of global OEF among the three groups. (b) Global OEF increases with age. Each symbol represents one participant.

OEF was 37.70 ± 5.09 % in the uninfected control controls, 37.02 ± 5.05 % in the non-hospitalized neuro-PCC participants and 42.40 ± 5.40 % in the hospitalized neuro-PCC participants. Figure 1a shows the comparisons of OEF between the neuro-PCC groups and the uninfected-control group. Linear regression analysis of all participants revealed a significant group effect in OEF (p=0.046), after accounting for age and sex. Specifically, participants with neuro-PCC that were previously hospitalized because of SARS-CoV-2 infection showed higher OEF compared to the uninfected controls (p=0.032), as well as those with neuro-PCC not hospitalized due to SARS-CoV-2 infection (p=0.015). The non-hospitalized neuro-PCC participants were not different from the uninfected controls. In addition to the group effect, OEF varied by age (p<0.001, Figure 1b) but not by sex (p=0.177).

**Figure 2:**
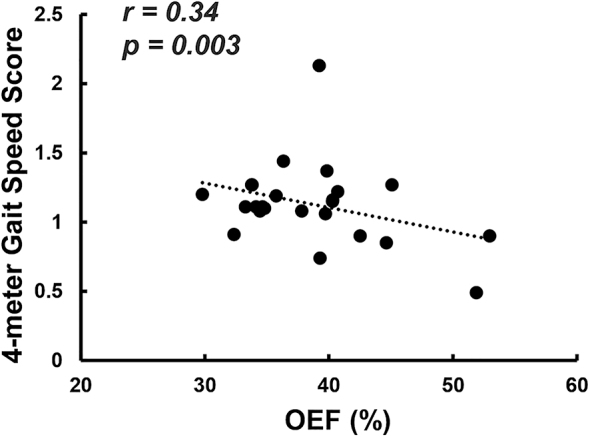
Scatter plot between global OEF and 4-m gait speed score. Each dot represents one participant with neuro-PCC.

Among the 25 participants with neuro-PCC who were evaluated with the NIH Toolbox^®^ (NIHTB) Motor Battery, after regressing out the age effect, OEF was inversely associated with the 4-m walk gait speed score (β=−0.03, r=0.34, p=0.003, Figure 2). Participants with neuro-PCC who had higher OEF showed poorer locomotor function. We also quantified the white matter hyperintensity (WMH) volume from the fluid-attenuated inversion recovery (FLAIR) images of each participant with neuro-PCC, but no association was found between WMH volume and OEF (p=0.11), or between WMH volume and the 4-m walk gait speed score (p=0.28).

## Discussion

In this study, we used a T2-relaxation-under-spin-tagging (TRUST) MRI technique to quantify global OEF in participants with neuro-PCC after SARS-CoV-2 infection. Participants with neuro-PCC who were previously hospitalized for SARS-CoV-2 infection had higher OEF compared to both the uninfected controls and participants with neuro-PCC who were not hospitalized. Higher OEF was further found to be associated with poorer motor function as measured by 4-m walk gait speed in participants with neuro-PCC.

Mounting evidence suggests that dysfunction in cerebral microcirculation plays a major role in the pathogenesis of neuropsychiatric complications in COVID-19 patients with PCC [5, 18]. SARS-CoV-2 can enter the brain via binding of its spike protein to the angiotensin-converting enzyme (ACE) 2 receptors, which are widely located, especially on the vascular endothelial cells [5]. Multifocal vascular damage as determined by leakage of serum proteins into the brain parenchyma was reported by an autopsy study of the brains of patients who died after SARS-CoV-2 infection [18]. More recently, chronic blood-brain barrier (BBB) breakdown as measured by increased BBB-permeability to water molecules in COVID-19 survivors who had a severe acute infection was also found with a novel MRI technique [19]. Damages to the brain’s microvasculature result in diminished cerebral perfusion [6, 7] and consequently insufficient oxygen supply to the brain, which in turn may cause chronic brain damage. The relatively higher global OEF in the participants with neuro-PCC who were hospitalized due to SARS-CoV-2 infection supports our hypothesis that the insufficiency of oxygen supply relative to the demand might contribute to symptoms associated with neuro-PCC. This finding is consistent with another MRI study in a different cohort of COVID-19 patients, that also found abnormally elevated OEF and decreased CBF in the frontal lobes of 7 COVID-19 survivors who were critically ill and admitted to intensive care unit (ICU) because of SARS-CoV-2 infection [20]. In our study, while higher global OEF was found primarily in participants with neuro-PCC who were hospitalized, the OEF in the neuro-PCC participants who were not hospitalized were relatively normal. These findings suggest that the long-lasting alteration in oxygen homeostasis may only be present in more severe cases of SARS-CoV-2 infection. Currently, we are conducting a follow-up study to investigate whether the elevated OEF would normalize 1-year after the initial MRI in our participants with neuro-PCC who were hospitalized.

The age-dependent increase in OEF in our participants is consistent with prior studies [21, 22]. The increase of OEF with age is thought to reflect age-related compensation in brain function during normal aging. The elevated OEF in our hospitalized neuro-PCC participants remained significant after we co-varied for age. Therefore, this elevated OEF can be attributed to microvascular damage-related alteration in oxygen homeostasis instead of age-related compensation. The elevated OEF in the hospitalized neuro-PCC participants likely reflect the compensatory mechanisms in the brain similar to the age-related increases in OEF due to compensatory processes. The compensatory increases in OEF may also be due to cerebral hypoperfusion, which is consistent with a recent cerebral perfusion MRI study that found hypoperfusion [7] as well as a ^18^F-FDG PET study that found hypometabolism in the brains of patients with long COVID [23].

In our study, all participants with neuro-PCC were evaluated with the NIH Toolbox^®^ (NIHTB) Cognitive Battery and Motor Battery [12]. We previously found that the neuro-PCC participants were slower on dexterity (Pegboard dominant hand) and the 4-m walk gait speed, and they also had poorer performance on the 2-min walk, relative to the uninfected controls [24]. The association of higher OEF with slower locomotion on the 4-m walk in the neuro-PCC participants further indicates that the elevated OEF is a pathological process. These findings also demonstrate the sensitivity of OEF as a potential biomarker for detecting and quantifying neuropsychiatric symptoms associated with neuro-PCC in COVID-19 participants.

This study has several limitations. First, we only evaluated global OEF which lacks spatial resolution. Thus, it is unclear whether the elevated OEF is present throughout the brain or only in a few brain regions. Therefore, future study should include an improved MRI technique that can assess regional OEF [25]. Second, we did not conduct standardized cognitive tests on the healthy participants in the current study because the primary goal of this study was to assess whether participants with neuro-PCC exhibited altered oxygen utilization in their brains. Future studies should include uninfected healthy controls who will be evaluated with the same NIH Toolbox Cognitive Battery and Motor Battery as the neuro-PCC participants. Third, we did not perform antibody tests in the healthy controls; therefore, we could not ascertain that none of our control participants had prior asymptomatic SARS-CoV2 infections. The serologic antibody tests for past infection were unavailable or less accessible during this project period. Lastly, our sample size for the neuro-PCC participants, especially the hospitalized participants, is relatively small. A larger sample size including more hospitalized participants is needed to further verify the findings of this study.

## Conclusions

Participants with more severe neuro-PCC, requiring hospitalization, had elevated oxygen extraction suggesting an insufficiency in oxygen supply relative to the demand. The elevated OEF, indicating a compensatory process that was insufficient to maintain normal locomotion, and might have contributed to their slower locomotion. Cerebral oxygen extraction measurement is a potential marker to evaluate neuro-PCC in future studies.
